# A Prospective Study on Long-Term Clinical Outcomes of Patients With Lupus Nephritis Treated With an Intensified B-Cell Depletion Protocol Without Maintenance Therapy

**DOI:** 10.1016/j.ekir.2021.01.027

**Published:** 2021-02-03

**Authors:** Dario Roccatello, Savino Sciascia, Carla Naretto, Mirella Alpa, Roberta Fenoglio, Michela Ferro, Giacomo Quattrocchio, Elena Rubini, Elnaz Rahbani, Daniela Rossi

**Affiliations:** 1 CMID-Nephrology and Dialysis Unit (ERK-net Member), Center of Research of Immunopathology and Rare Diseases, Coordinating Center of the Network for Rare Diseases of Piedmont and Aosta Valley, Department of Clinical and Biological Sciences, University of Turin and S. Giovanni Bosco Hub Hospital, Turin, Italy

**Keywords:** B-cell depletion therapy, lupus nephritis, rituximab, systemic lupus erythematosus

## Abstract

**Background:**

We aimed to investigate the safety and efficacy of an intensified B-cell depletion induction therapy (IBCDT) without immunosuppressive maintenance regimen compared with standard of care in biopsy-proven lupus nephritis (LN).

**Methods:**

Thirty patients were administered an IBCDT (4 weekly rituximab [RTX] 375 mg/m^2^ and 2 more doses after 1 and 2 months; 2 infusions of 10 mg/kg cyclophosphamide [CYC], 3 methylprednisolone pulses), followed by oral prednisone (tapered to 5 mg/d by the third month). No immunosuppressive maintenance therapy was given. Thirty patients matched for LN class and age were selected as controls: 20 received 3 methylprednisolone pulses days followed by oral prednisone and mycophenolate mofetil (MMF) 2 to 3 g/d, whereas 10 were given the Euro Lupus CYC. MMF (1-2 g/daily) or azathioprine (AZA, 1-2 mg/kg/day) were given for > 3 years as a maintenance therapy.

**Results:**

At 12 months, complete renal remission was observed in 93% of patients on IBCDT, in 62.7% on MMF, and in 75% on CYC (*P* = 0.03); the dose of oral prednisone was lower in the IBCDT group (mean ± SD 2.9 ± 5.0 mg/dl) than MMF (10.5 ± 8.0 mg/d, *P* < 0.01) or CYC group (7.5 ± 9.0 mg/d, *P* < 0.01). Mean follow-up after treatment was 44.5 months (interquartile range [IQR] 36–120 months), 48.6 months (IQR 36–120 months), and 45.3 (IQR 36–120 months) for IBCDT, MMF, and CYC, respectively. At their last follow-up visit, we observed no significant differences in proteinuria and serum creatinine, nor in the frequency of new flares among the 3 groups.

**Conclusion:**

In biopsy-proven LN, the IBCDT without further immunosuppressive maintenance therapy was shown to be as effective as conventional regimen of MMF or CYC followed by >3-year maintenance either MMF or AZA regimen. Moreover, the use of IBCDT was associated with a marked reduction of glucocorticoid cumulative dose.

## Graphical abstract

**Figure:**
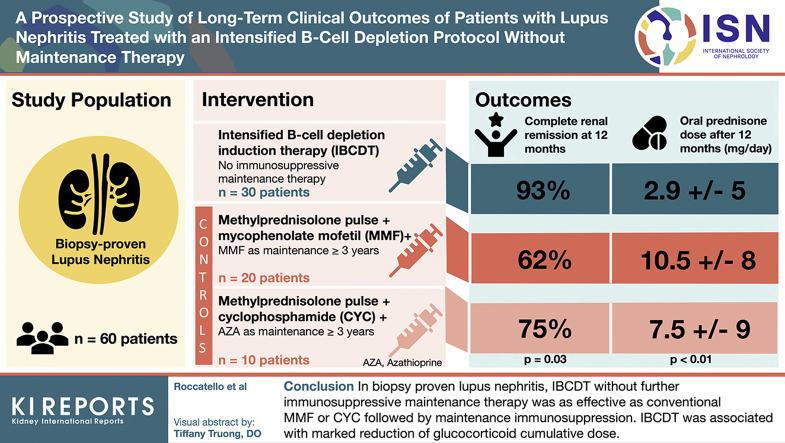

Systemic lupus erythematosus (SLE) is characterized by a heterogeneous spectrum of manifestations, varying in terms of clinical features and severity. LN can be observed in up to half of the patients with SLE and its occurrence affects morbidity and mortality.^1^, ^2^, ^3^, ^4^, ^5^, ^6^ We experienced dramatic changes in the management of LN over the past decades. The main cornerstones of LN treatment rely on the optimization of CYC protocols, the introduction of novel therapeutic options such MMF, and, more recently, calcineurin inhibitors (e.g., tacrolimus). Similarly, there has been a continuous effort to design therapeutic strategies aimed at reducing the steroid dose.^7^ The recently disseminated European League Against Rheumatism recommendations support the use of MMF/mycophenolic acid and low-dose i.v. CYC (EUROLUPUS scheme) as the treatments of choice for the induction of remission. MMF and azathioprine are the recommended options as maintenance therapy.^8^

However, regardless of the improvement in overall mortality and morbidity, conventional immunosuppression is still associated with a high incidence of side effects, and the search for alternative therapeutic options remains a priority.^7^

Despite the disappointing results of randomized controlled trials,^9^ targeting B cells remains an attractive option in patients with LN.^10^

We previously reported the promising outcome of patients with severe SLE treated with an IBCDT protocol, including RTX, CYC, and methylprednisolone pulses.^11^^,^^12^ IBCDT proved to be safe, well-tolerated, and effective both for the induction of remission and for long-term remission maintenance despite the absence of further immunosuppressive maintenance therapies.^11^^,^^12^ The aim of this prospective study was to investigate the long-term safety and efficacy of IBCDT in patients with active LN as compared with conventional immunosuppressants (CYC pulses or MMF) followed by a 3-year maintenance MMF regimen.

## Methods

### Patients

Sixty patients with active, biopsy-proven LN (48 women [80%]; 58 [97%) Caucasian, 2 (3%) Black) followed at the CMID-Nephrology and Dialysis Unit and Center of Research of Immunopathology and Rare Diseases and Dialysis, S. Giovanni Bosco Hospital, Turin, Italy (2005–2018) were enrolled in this study. All patients fulfilled the American College of Rheumatology classification criteria for SLE.^13^ Median age at LN diagnosis was 42.3 years (range 23–65). SLE was diagnosed a median of 13.5 months (0–36) before the diagnosis of LN was made. All patients underwent a renal biopsy. Clinical and histological characteristics are shown in Table 1. Renal biopsy classification was carried out as recommended by the International Society of Nephrology 2003/Renal Pathology Society.^14^ Thirty-two patients (53%) were included in the study when LN was diagnosed, 22 (37%) patients entered the study when a LN flare occurred, and 6 (10%) patients entered due to refractory renal disease.

**Table 1:** Baseline Characteristics

	IBCDT *n* = 30	MMF *n* = 20	CYC *n* = 10	*P*
Class IV,^a^*n* (%)	10 (33.3)	6 (30)	4 (40)	1
Class III/V, *n* (%)	10 (33.3)	6 (30)	4 (40)	1
Class V, *n* (%)	10 (33.3)	8 (40)	2 (20)	1
sCr mg/dl, median (IQR)	0.91 (0.6–1.5)	0.83 (0.6–1.4)	0.98 (0.6–1.7)	0.56
C3/C4 mg/dl, median (IQR)	61 (40–99)/13 (4–16)	59 (45–101)/12 (5–21)	62 (44–93)/11 (4–17)	0.63
Proteinuria g/24 h, median (IQR)	5.0 (3.7–9.1)	4.6 (3.0–8.6)	5.2 (3.7–9.9)	0.45
Anti-dsDNA IU median (IQR)	176 (37–212)	145 (31–189)	181 (41–199)	0.39
Albuminemia g/dl, median (IQR)	3.0 (2.8–3.8)	2.89 (2.6–3.7)	3.0 (2.8–3.5)	0.57
Urinary red blood cells, median (IQR)	37 (10–100)	51 (5–100)	41 (5–100)	0.45
SLEDAI, median (IQR)	21.5 (14–25)	18 (14–24)	21 (16–25)	0.45
First-line treatment,^a^*n* (%)	15 (50)	10 (50)	5 (50)	1
New flare,^b^*n* (%)	9 (30)	6 (30)	3 (30)	1
Refractory LN (%)	6 (20)	4 (20)	2 (20)	1

CYC, cyclophosphamide; dsDNA, double-stranded DNA; IBCDT, intensified B-cell depletion induction therapy; IQR, interquartile range; LN, lupus nephritis; MMF, mycophenolate mofetil; sCr, serum creatinine; SLEDAI, Systemic Lupus Erythematosus Activity Index.

a Patients with no sign of active LN in the previous 12 months.

b Rapidly progressive glomerulonephritis, with >50% florid crescents feature in 2 cases treated with IBCDT and 1 case with CYC.

For this study, controls were selected among the cohort of patients being treated for active LN at the Center and were matched 1:1 based on LN histology, age, and indication for treatment (newly diagnosed, new renal flare or refractory renal disease, according to Kidney Disease: Improving Global Outcomes definitions).^15^

The clinical outcome of 12 of these patients treated with IBCDT has previously been reported.^12^

### Ethical Approval

All subjects provided written consent according to the Declaration of Helsinki. This study was performed according to the local rules of off-label therapy in Piedmont (Northwest Italy).

### Therapeutic Schedules

#### IBCDT

Thirty patients received IBCDT according to the following scheme. RTX was administered intravenously as previously described,^11^^,^^12^ at a dosage of 375 mg/m^2^ on days 2, 8, 15, and 22. Two more doses were administered 1 and 2 months following the last weekly infusion. This treatment was combined with 2 pulses of 10 mg/kg CYC (reduced, if needed, according to renal impairment) at days 4 and 17, and 3 i.v. pulses of 15 mg/kg (days 1, 4, and 8) methylprednisolone followed by oral prednisone, 50 mg for 2 weeks tapered to 5 mg in 3 months.

#### Controls

The patients were given 1 i.v. pulse of methylprednisolone (1000 mg if body weight >50 kg, 500 mg if <50 kg) for 3 consecutive days, followed by prednisone 0.5 to 0.75 mg/kg per day for 1 month, then tapered according to the following scheme: 0.4 to 0.5 mg/kg per day for 1 month, then 0.25 to 0.35 mg/kg per day for 1 month, followed be a reduction of 2.5 to 5 mg every 14 days until a dosage of 5.0 to 7.5 mg daily. Twenty patients received MMF 2 to 3 g/d, and 10 patients were administered 1 i.v. pulse of CYC every fortnight for a total of 6 administrations (500 mg for a total of 3000 mg).^15^ After 3 months of treatment, the patients who had previously been treated with CYC received azathioprine 1 to 2 mg/kg per day as maintenance therapy in addition to prednisone. Patients who were induced with MMF continued MMF at a dosage of 1 to 2 g/d. Maintenance therapy lasted a median of 5.5 years (range 3.7–7.0 years).

### Study Outcome Measurements

#### Primary Outcomes

Primary outcomes were (i) complete renal remission at 12 months, and (ii) time free from flares during follow-up.

#### Secondary Outcomes

Secondary outcomes included (i) changes in immunological and biochemical parameters, (ii) safety profile of the regimens and rate of side effects, (iii) steroid-sparing effect, (iv) response as assessed by the Systemic Lupus Erythematosus Activity Index-2K, and (v) rate of renal flares.

#### Definitions

European League Against Rheumatism/European Dialysis and Transplant Association response to therapy was defined as follows^16^:

• Complete renal response: proteinuria <0.5 g/24 hours, normal or near-normal estimated glomerular filtration rate (within 10% of normal estimated glomerular filtration rate if previously abnormal).
• Partial renal response: ≥50% reduction in proteinuria to subnephrotic levels (<3.5 g/24 hours), and normal or near-normal estimated glomerular filtration rate.
• No renal response: all the other cases.

Overall disease activity was assessed by Systemic Lupus Erythematosus Activity Index-2K.^17^

• Severe infection: (i) deep tissue (invasive) infection requiring i.v. or oral antibiotics used to treat infection; (ii) any infection requiring hospitalization, if outpatient at onset; (iii) any infection leading to need for oxygen, pressors, or fluids to support blood pressure or intubation; (iv) pulmonary nodules that decrease in size after a minimum 4-week course of antifungal medications active against Aspergillus; (v) any Bacteremia, catheter-related bloodstream infection; (vi) any infection that requires adjunctive surgical intervention; (vii) disseminated or complicated zoster (i.e., ophthalmic).

### Statistical Analysis

For the comparison of variables at baseline and follow-up, Student's *t*-test was used for normally distributed parameters and the nonparametric Mann–Whitney test for non-normally distributed parameters. Correlations were calculated and significance was determined by Fisher's exact test. Multivariable logistic regression analysis was used to identify any independent predictors of flare. Kaplan–Meier hazard plots were constructed for time to renal flare. For these analyses, the SPSS (IBM Corporation, Armonk, NY) software was used; *P* < 0.05 was considered statistically significant.

## Results

The main characteristics of the patients at baseline are shown in Table 1. When comparing patients treated with IBCDT and those who received MMF or CYC pulses to baseline, no significant differences in terms of serum creatinine, proteinuria, or number of red blood cells in urinary sediment scores were observed.

At 12 months, complete renal response (CRR) had been achieved in 28 (93%) patients treated with IBCDT, in 13 (65%) on MMF, and in 7 (70%) on CYC; IBCDT was found to be associated to a higher rate of response when compared with the other regimens (*P* = 0.03) (Table 2). Partial renal response was observed in 2 (7%) patients treated with IBCDT, in 4 patients on MMF (20%), and in 3 (30%) in the CYC group. No response was observed in 3 patients on MMF (15%). Time to CRR did not statistically differ among the 3 groups (Table 2).

**Table 2:** Clinical and Serological Profile at 12 Months

	IBCDT *n* = 30	MMF *n* = 20	CYC *n* = 10	*P*
sCr	0.89 (0.6–1.4)	0.85 (0.6–1.4)	0.97 (0.6–1.7)	0.68
ESR, median (IQR)	10.2 (7–27)	14(8–28)	21 (8–30)	0.71
Proteinuria g/24 h, median (IQR)	0.46 (0.37–0.50)	0.71 (0.30–0.91)	0.51 (0.33–0.82)	0.78
C3/C4 mg/dl, median (IQR)	97 (79–120)/20 (11–37)	95 (81–120)/21 (13–40)	99 (80–120)/20 (10–40)	0.74
SLEDAI median, (range)	4 (1–5)	6 (2–8)	4 (2–8)	0.57
Complete renal response, *n* (%)	28 (93)	13 (65)	7 (70)	**0.03**
Partial renal response, *n* (%)	2 (7)	4 (20)	3 (30)	0.15
No renal response, *n* (%)	0 (0)	3 (15)	0 (0)	0.37
Any renal response, *n* (%)	30 (100)	17 (85)	10 (100)	0.32
Time to complete renal response, median months (IQR)	9 (6–11)	8 (6–11)	7(6–11)	0.87

CYC, cyclophosphamide; ESR, erythrocyte sedimentation rate; IBCDT, intensified B-cell depletion induction therapy; IQR, interquartile range; MMF, mycophenolate mofetil; sCR, serum creatinine; SLEDAI, Systemic Lupus Erythematosus Activity Index

Twelve months after baseline assessment, none of the patients in the IBCDT group had serum albumin levels <3.5 g/dl, whereas this abnormality was observed in 4 patients on MMF and in 2 in the CYC group, respectively. Immunological and renal function parameters at 12 months are shown in Table 2.

We observed a progressive reduction in the Systemic Lupus Erythematosus Activity Index score in all groups, although the decline was more marked in the IBCDT group. A gradual improvement in the articular and muco-cutaneous symptoms was observed in all groups. Three patients treated with CYC showed persistent haematological manifestations, mainly anemia (low-to-moderate).

Complete peripheral blood B-cell depletion was achieved in all patients who received IBCDT. The CD20+/CD19+ B cells were assessed every 3 months for the first 18 months and every 6 months thereafter. The CD20+/CD19+ B cells were detectable in the circulation after a median of 12.6 months (11–19 months). Remarkably, when assessed 2 years after the beginning of therapy with IBCDT, CD20+ B-cell count was still lower than baseline (*P* > 0.05).

Ig levels were evaluated every 3 months for the first 18 months and every 6 months thereafter, with no patients experiencing moderate to severe hypogammaglobulinemia (level IgG levels below 600 mg/dl). Patients were regularly monitored for osteopenia/osteoporosis with regularly scheduled bone density scan (dual-energy X-ray absorptiometry scan) (Table 3).

**Table 3:** Glucocorticoids-related Comorbidities Assessed at End of Follow-up

	IBCDT *n* = 30	MMF *n* = 20	CYC *n* = 10	*P*
Osteopenia/osteoporosis, *n* (%)^a^	2 (7)	4 (20)	2 (20)	0.31
Diabetes, *n* (%)	1 (3)	4 (20)	1 (10)	0.16
Glaucoma, *n* (%)	1 (3)	4 (20)	3 (30)	0.055

	IBCDT *n* = 30	MMF and CYC *n* = 30	*P*
Osteopenia/osteoporosis, *n* (%)^a^	2 (7)	6 (20)	0.12
Diabetes, *n* (%)	1 (3)	5 (17)	0.08
Glaucoma, *n* (%)	1 (3)	7 (23)	**0.022**

CYC, cyclophosphamide; IBCDT, intensified B-cell depletion induction therapy; MMF, mycophenolate mofetil.

a As assessed by bone density scan (dual-energy X-ray absorptiometry scan).

### Side Effects

No severe adverse events were observed in the IBCDT group (2 cases of infusion speed-related bradycardia), and no severe infections were reported during the follow-up. One patient treated with IBCDT experienced 1 episode of urinary tract infection after 24 weeks from treatment. Four patients treated with MMF experienced gastrointestinal symptoms, mainly diarrhea, requiring tapering of the MMF dosage (2 cases) or the shift to mycophenolic acid (2 cases). One patient developed self-limiting herpes zoster after 8 months from the beginning of the therapy. Transient leukopenia was observed in 2 patients in the CYC group (after 3 months from the beginning of the therapy). No severe case of neutropenia was observed in the follow-up. One patient had 1 episode of urinary tract infection after 24 months from the first dose of CYC. One patient in the CYC group developed transient amenorrhea.

### Steroid-sparing Effects

One month after the beginning of induction therapy, patients on IBCDT were being administered a significantly lower dose of steroids (prednisone mean dose 25.0 ± 5.0 mg/d) as compared with those on MMF (40.5 ± 15.7 mg/d, *P* < .01) or on CYC (35.4 ± 41.0 mg/d, *P* < 0.01). The trend was maintained after the 12th month, with patients in the IBCDT group receiving lower doses of steroids (prednisone mean dose 2.9 ± 5.0 mg/dl) than those on MMF (10.5 ± 8.0 mg/d, *P* < 0.01) or CYC (7.5 ± 9.0 mg/d, *P* < 0.01).

We observed an overall trend in lower rate of glucocorticoids-related comorbidities in the IBCDT (Table 3), reaching statistical significance for the occurrence of glaucoma at the end of follow-up.

### Patient Outcome Beyond Month 12

**Figure 1:**
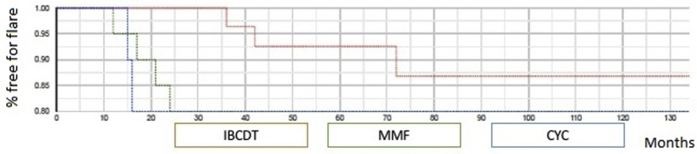
Time free from flares curves in patients treated with IBCDT, MMF, or CYC. CYC, cyclophosphamide; IBCDT, intensified B-cell depletion induction therapy; MMF, mycophenolate mofetil.

Mean follow-up after treatment was 44.5 months (IQR 36–120 months), 48.6 months (IQR 36–120 months), and 45.3 (IQR 36–120 months) for IBCDT, MMF, and CYC, respectively, and did not differ among the groups (*P* = 0.56). None of the patients entered end-stage renal disease and none died. When comparing the outcomes of the 3 groups at the last observation available, we observed no statistically significant differences in terms of serum creatinine and proteinuria levels. Conversely, we found a higher rate of flares in patients who received MMF (4, 20%) or CYC (2, 20%) compared with IBCDT (3, 10%), albeit it did not reach statistical significance (*P* = 0.55). Mean time to flare was 57.9 ± 32.44 in the IBCDT, 57.3 ± 37.52 in the MMF, and 51.67 ± 62.64 in the CYC groups, respectively. In the IBCDT group, 3 patients experienced a flare after 36, 42, and 72 months, respectively. Following re-treatment, they showed CRR over a median follow-up of 92 months (Figure 1).

## Discussion

B cells play a key role in the pathogenesis of SLE, as they are involved in the production of autoantibodies targeted against a heterogeneous pool of self-antigens, therefore leading to inflammation and organ damage. Intriguingly, a growing body of evidence now supports the hypothesis that B cells play a pivotal role beyond the production of autoantibodies, including the activation dendritic cells and the modulation of T-cell functions (tolerance, induction of autoreactive memory T cells, activation of Th1 and Th17 cells). More recently, the modulatory effect on follicular B-helper T cells has also been discussed in the pathogenesis of SLE.^17^, ^18^, ^19^ Based on the previously mentioned points, depletion of B cells might appear to be a logical therapeutic target that could provide clinical benefits in SLE, assuming that the interventions achieve deep and sustained elimination of pathogenic B cells.

We showed IBCDT to be a safe and effective therapeutic strategy in the management of patients with severe LN.

As compared with standard treatments, IBCDT was at least as safe and effective as standard MMF or CYC-based regimens in inducing clinical remission. Most importantly, despite the absence of immunosuppressive maintenance regimen, IBCDT proved to have a long-term efficacy profile similar to standard induction-maintenance therapy. Indeed, among the patients who achieved CRR after 12 months, 89% (25/28) remained in remission after 1 cycle of IBCDT without any further immunosuppressive maintenance treatment, and these patients did not relapse. These data confirm our previous results in cases of refractory SLE^10^ and severe cases of SLE.^11^

There are several points to be discussed when critically analyzing our results. First, to validate patients’ matching, all our patients receive a renal biopsy close to treatment beginning. Second, one strength of our treatment strategy is related to the relatively short time of immunosuppression that patients treated with IBCDT are exposed to, which remarkably reduces the risk of possible adverse effects related to the prolonged use of steroids and CYC.^12^ Long-lasting remission without immunosuppressive maintenance therapy makes the IBCDT regimen particularly appealing in low-compliance patients. Pragmatically, we observed that up to 93% of patients treated with IBCDT achieved CRR at 12 months, with an overall 10% rate of flares during follow-up. Ninety percent of patients experienced a symptom-free period without urinary abnormalities for more than 120 months.

Third, it is worth mentioning that IBCDT allowed the reduction of steroid burden without a loss in efficacy, as compared with conventional regimens. In fact, as of the third month of therapy, patients were receiving a median dose of prednisone <5 mg, which was significantly lower than what was being administered in conventional protocols.

Moreover, no safety signals were observed compared with studies with a comparable long-term follow-up in terms of incidence of adverse events (as reviewed in Roccatello *et al.*^12^), particularly when referring to severe infections (not observed in our cohort).

Based on the previously mentioned items, perhaps we should reconsider our view on RTX, regardless of the disappointing results of randomized controlled trials, especially the LUNAR trial.^9^ Indeed, there are several points to be considered when interpreting data from patients of LUNAR study.

The first point is the background therapy. RTX was given as an add-on therapy to gold standard treatments, limiting the possibility of exploring the differences in clinical response between nonrefractory patients and refractory patients. It is worth mentioning that the subset of patients who were refractory to previous therapy represents the focus of most of the available literature involving uncontrolled open studies investigating the use of RTX (as discussed in Cervera *et al.*^7^). Indeed, Black and Hispanic individuals, who are known to be more resistant to therapy than Caucasian individuals, showed a better outcome when receiving RTX in the LUNAR trial.^9^ Another point is the relatively short follow-up of the LUNAR study, perhaps too short to demonstrate any significant differences between the arms. Finally, the clinical outcome parameters used in the LUNAR study were very stringent when compared with other studies,^20^ and did not include surrogate markers of efficacy, such as the steroid dose-sparing effect. Nevertheless, the LUNAR trial provided a considerable body of scientific information and promoted a critical debate on the characteristics of patients who might benefit from RTX treatment. The most relevant is the extent of RTX-induced peripheral blood B-cell depletion that is needed to achieve CRR in LUNAR patients. This aspect has been reevaluated recently. Incomplete peripheral blood B-cell depletion might correlate with the inability to reduce tubulointerstitial lymphoid aggregates in the kidney, leading to an inadequate response to treatment.^21^ Complete clinical response at week 78 has been associated with complete depletion of CD19 peripheral cells (i.e., # 0) and with a longer duration of peripheral depletion (>71 days). Our results are perfectly in line with these observations. Indeed, all our IBCDT-treated patients who had CRR at 12 months achieved a complete peripheral depletion (i.e., CD19 # 0).

### Limitations of the Study

The open, nonblinded design of our study and the relatively limited number of enrolled subjects might represent limitations when interpreting our results. These aspects are counterbalanced by the fact that our data are supported by a long-term follow-up in a cohort of real-life, biopsy-proven LN patients. Moreover, this is the first study in which the efficacy and safety profile of a combined IBCDT regimen was compared with standard CYC- or MMF-based protocols.

## Conclusions

Although a large-scale randomized controlled trial is warranted to confirm our findings, in this prospective study we showed that the IBCDT regimen is at least as effective as MMF or CYC pulses in inducing remission in patients with active LN. Furthermore, patients treated with IBCDT achieved at least comparable results in terms of efficacy and long-lasting remission as standard regimens. This was maintained with minimal doses of prednisone starting from the end of the third month after IBCDT without further immunosuppressive maintenance therapy. This approach prevented prolonged immunosuppression and remarkably reduced the risk of steroid-related adverse effects.

## Disclosure

The authors declared no competing interests.
